# Nanomaterials in the Management of Gram-Negative Bacterial Infections

**DOI:** 10.3390/nano11102535

**Published:** 2021-09-28

**Authors:** Mahmood Barani, Mahira Zeeshan, Davood Kalantar-Neyestanaki, Muhammad Asim Farooq, Abbas Rahdar, Niraj Kumar Jha, Saman Sargazi, Piyush Kumar Gupta, Vijay Kumar Thakur

**Affiliations:** 1 Medical Mycology and Bacteriology Research Center, Kerman University of Medical Sciences, Kerman 7616913555, Iran; Mahmoodbarani7@gmail.com (M.B.); d.kalantar@kmu.ac.ir (D.K.-N.); 2 Department of Pharmacy, Faculty of Biological Sciences, Quaid-i-Azam University, Islamabad 45320, Pakistan; mz1712@yahoo.com; 3 Department of Medical Microbiology (Bacteriology and virology), Afzalipour Faculty of Medicine, Kerman University of Medical Sciences, Kerman 7616913555, Iran; 4 Faculty of Pharmacy, Department of Pharmaceutics, The University of Lahore, Lahore 54000, Pakistan; pharma1154@yahoo.com; 5 Department of Physics, University of Zabol, Zabol 9861335856, Iran; 6 Department of Biotechnology, School of Engineering and Technology, Sharda University, Greater Noida 201310, India; nirajkumarjha2011@gmail.com; 7 Cellular and Molecular Research Center, Research Institute of Cellular and Molecular Sciences in Infectious Diseases, Zahedan University of Medical Sciences, Zahedan 9816743463, Iran; sgz.biomed@gmail.com; 8 Department of Life Sciences, School of Basic Sciences and Research, Sharda University, Greater Noida 201310, India; 9 Biorefining and Advanced Materials Research Center, SRUC, Edinburgh EH9 3JG, UK; 10 Department of Mechanical Engineering, School of Engineering, Shiv Nadar University, Greater Noida 201314, India; 11 School of Engineering, University of Petroleum & Energy Studies (UPES), Dehradun 248007, India

**Keywords:** *Escherichia coli*, nanotechnology, infection, diagnosis, treatment

## Abstract

The exploration of multiplexed bacterial virulence factors is a major problem in the early stages of *Escherichia coli* infection therapy. Traditional methods for detecting *Escherichia coli* (*E. coli)*, such as serological experiments, immunoassays, polymerase chain reaction, and isothermal microcalorimetry have some drawbacks. As a result, detecting *E. coli* in a timely, cost-effective, and sensitive manner is critical for various areas of human safety and health. Intelligent devices based on nanotechnology are paving the way for fast and early detection of *E. coli* at the point of care. Due to their specific optical, magnetic, and electrical capabilities, nanostructures can play an important role in bacterial sensors. Another one of the applications involved use of nanomaterials in fighting microbial infections, including *E. coli* mediated infections. Various types of nanomaterials, either used directly as an antibacterial agent such as metallic nanoparticles (NPs) (silver, gold, zinc, etc.), or as a nanocarrier to deliver and target the antibiotic to the *E. coli* and its infected area. Among different types, polymeric NPs, lipidic nanocarriers, metallic nanocarriers, nanomicelles, nanoemulsion/ nanosuspension, dendrimers, graphene, etc. proved to be effective vehicles to deliver the drug in a controlled fashion at the targeted site with lower off-site drug leakage and side effects.

## 1. Introduction

*Escherichia coli* (*E. coli*) is a gram-negative bacteria and causative agent of many infectious diseases in humans. Many bacterial infections such as urinary tract infections, bloodstream infections, pneumonia, surgical site infections [1,2,3], bacterial sepsis [4,5], and neonatal bacterial meningitis are mainly produced by *E. coli* [6].

The Gram-negative bacteria are characterized by their cell envelopes, which are composed of a thin peptidoglycan cell wall sandwiched between an inner cytoplasmic cell membrane and a bacterial outer membrane (OM) [7,8]. The OM is an additional protection layer that prevents several substances from entering the bacterium. Nevertheless, OM comprises channels named porins, which allow access to numerous molecules such as drugs [9]. The OM of Gram-negative bacteria is the leading cause of resistance for a wide range of antibiotics such as β-lactams, quinolones, and other antibiotics [10]. Most antibiotics must pass through OM for effective targeting [11]. Hydrophobic molecules can penetrate through the diffusion pathway; in contrast, hydrophilic antibiotics, including β-lactams can pass via porins. Any variation in the OM by Gram-negative bacteria, including mutations in porins, can cause resistance [12].

The use of antibiotics is an efficient, prevailing, and the utmost method for treating *E. coli* infections. However, huge numbers of drug-resistance strains have appeared due to antibiotics misuse in the last 50 years [13,14]. Furthermore, the inappropriate and overuse of antimicrobial agents has increased pathogens and humans’ resistance [15]. Numerous antibacterial agents such as ampicillin, cotrimoxazole, azithromycin, and gentamicin for *E. coli* therapy have been revealed to stimulate the Shiga toxin release from *E. coli* [16]. In addition, the antibodies treatment is an effective method for deactivating the virulence factors and toxins from *E. coli* [17]. Still, the specificity of antibodies is a major challenge for treating *E. coli* infections using antibodies [18]. Vaccine therapy using inactivated *E. coli* has been used to robust the immune responses in humans. However, the short duration of the vaccine producing immunity against bacterial infections is a major drawback for treating *E. coli* [19]. Despite this, antibiotics-based therapy is still the main strategy against bacterial infections. There is a need to discover new antibacterial agents with new mechanisms to combat resistant bacterial strains [20].

Conventional methods have been used for the diagnosis of *E. coli* infections for several years, including enzyme-linked immune sorbent assay (ELISA) and polymerase chain reaction (PCR) [21]. The non-culturing approaches are conducted by staining the urine sample for the detection of bacterial infections, but these approaches are time-consuming with less precision value [22]. Meanwhile, the culturing method is one of the oldest techniques for detecting infectious bacteria. Few drawbacks accompany this method, e.g., preparation of individual culture medium to detect each microorganism in the sample for optimal growth [23]. PCR-based methods have been utilized for the identification and diagnosis of bacterial infections [24]. A multiplex PCR test has been established to recognize *E. coli* producing bacterial infections [25]. ELISA is also one of the molecular techniques widely used to detect bacterial components in the sample [26]. Nevertheless, the prolonged incubation period, extensive sample cleaning, and purification of biomolecules are major disadvantages of these methods [27,28]. To tackle limitations related to the approaches mentioned above, nanotechnology is a quick, efficient and versatile solution for treating and detecting bacterial infections [29]. Recently, numerous NPs, such as silver NPs, zinc oxide NPs, and cationic surfactant NPs, have been used for bacterial infection treatment [30,31,32]. The antibacterial potential of silver NPs generally depends on the particle size, shape and surface modification [33,34,35,36]. The loading of antibacterial moiety into the silver NPs also enhances its antimicrobial activity [37,38]. Zinc oxide is a multifunctional inorganic material that has been used widely in optoelectronic devices, textiles, cosmeceuticals, and most importantly, as an antibacterial agent [39]. The cationic surface NPs are positively charged and can kill bacteria by disrupting bacterial cell wall/membrane, generating free radicals [40].

Nanotechnology-based approaches such as gold NPs, silver NPs, magnetic NPs, and quantum dots (QDs) reveal selective target-binding characteristics [41,42,43,44,45,46,47,48,49,50,51,52,53,54,55,56]. These characteristics make them ideal candidates for the diagnosis and biosensing of *E. coli* infections [57,58,59]. The binding to specific ligands such as antibodies and enzymes for detecting bacterial infections is due to the surface properties of NPs. This boosts the specificity of the nanosensor being developed [60]. The entrapment of NPs into nanosensors also enhances the rapid detecting ability of the portable device. NPs, portable devices, nanotubes, nanowires, and nanomechanical devices are typical examples of functional probes for the detection and disinfection of pathogens and other contaminants in different mediums [61,62,63].

## 2. Detection of *E. coli* Infection

Specific and accurate identification of bacteria is a critical component of early illness diagnosis and surveillance, and it has the ability to avert outbreaks and the spread of devastating epidemics. It is also vital to quickly confirm the presence of these bacteria at low concentrations to prevent these diseases. As previously stated in the introduction, traditional approaches for detecting *E. coli* have significant disadvantages that restrict their implementation in real-world situations [64,65,66].

### 2.1. Nanotechnology Approaches for E. coli Detection

Early diagnosis of specific species of bacteria has numerous medical, environmental, and food safety uses [67]. NPs have shown exceptional abilities against various infections and are being exploited to build new devices and technology to help with this public health problem [68,69]. Since zoonosis is an existing thread, the focus is not confined to human diseases but also includes those that affect animals. For example, Stringer et al. developed an optical biosensor for the identification of respiratory syndrome and porcine reproductive virus, utilizing QDs and gold NPs [70]. For the diagnosis of viral and bacterial clinical infections, biosensors and nano-biosensors have been widely used. These tools are practical (smartphone-based nano-biosensor and POC ability) and rapid, and they are regarded as novel technologies that provide a replacement to the drawbacks of traditional investigative techniques. In this review, we discuss different nanomaterials (Gold NPs, Ag NPs, Carbon NPs, QDs, etc.) for effective detection of *E. coli* in wastewater, food, and the human body. Different nanomaterials used in the detection of *E. coli* are summarized in Table 1.

#### 2.1.1. Gold NPs

Due to its versatility in dimension and arrangement, such as circular, diamond, crystalline, triangular, and spiral geometries, gold NPs have been extensively used in *E. coli* recognition. In comparison to native Au, gold NPs have distinct physical, chemical, and electrical properties [94]. In this light, Li et al. developed a procedure for detecting the *E. coli* O157:H7 bacteria by using gold NP labelling, antibody affinity binding, and inductively coupled plasma mass spectrometry (ICPMS) [71]. The technique was capable of detecting as few as 500 *E. coli* O157:H7 cells in 1 mL of sample or 500 CFU mL^−1^ using the signal amplification ability of Au NPs and the perfect sensitivity of ICPMS. The assay had good specificity for *E. coli* O157:H7 in tests with non-pathogenic *E. coli* (DH5r, TCC35218, and ATCC25922). Each experiment took 40 min to complete. Demonstration of this assay for E. coli O157:H7 suggests its potential for detecting a variety of bacterial pathogens.

In another study, Wang et al. reported a quartz crystal microbalance (QCM) biosensor for *E. coli* O157:H7 DNA identification using nanogold modification and mass amplification [72]. 1,6-Hexanedithiol was applied to the Au surface of QCM and subsequently self-assembled to form a thiolated interface, enabling Au NPs anchoring inside the device. Thiolated single-stranded DNA (ssDNA) probes targeted the *E. coli* O157:H7 *eaeA* gene connected to the NP-modified electrode surface through the Au-SH binding. The outer nanogold was used as a mass amplifier to boost the signal, and the biotin-avidin system was used to bond with the target DNA. This biosensor identified target DNA corresponding to 2 × 10^3^ CFU mL^−1^ *E. coli* O157:H7, indicating that developing an appropriate and sensitive QCM biosensor for harmful bacteria diagnosis based on specific DNA analysis is feasible. In order to attract more attention, this DNA biosensor should be combined with some micro- and nano-fabrication techniques to realize more promising and practical applications, and more attention should be focused on the further improvement of the sensitivity and the shortening of analysis time.

**Figure 1:**
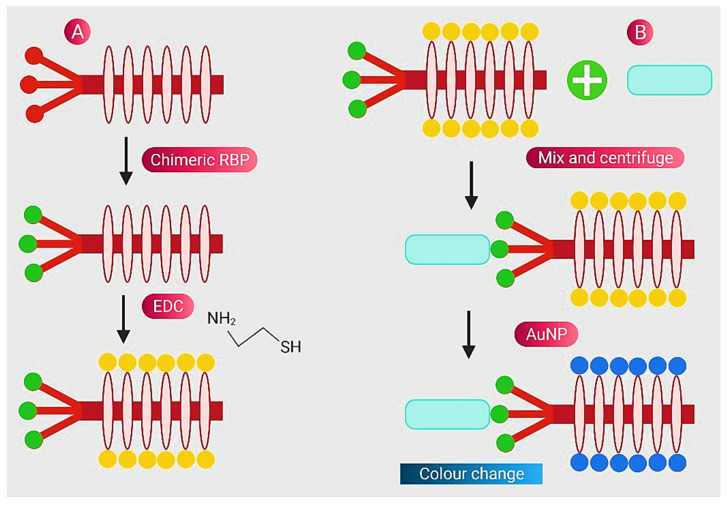
Schematic representation for chimeric phages and detection of pathogens (**A**) Gray: M13 phage, blue: foreign receptor-binding protein and yellow: thiolated phage by EDC chemistry (**B**) Blue rectangle: bacteria-infected medium, red: Au NPs and colour change (purple): attachment of Au NPs and thiolated phage. Reprinted from Ref. [73].

Based on the interaction between bacterial cells and the viruses that infect them (phages), Peng et al. provided a straightforward technique for identifying a range of bacterial species [73]. As phages are less expensive and more stable to storage and testing conditions than antibodies, they have previously been examined for bacterial identification [95]. This group modified phage M13 to express a receptor-binding protein from a phage that normally identifies the bacteria they want to kill. Thiolation of the altered phages enables Au NPs to attach to them, causing them to assemble on the phages and operate as a signal amplifier, resulting in an apparent colour change due to changes in surface plasmon resonance characteristics. Two strains of *E. coli*, the human infections *Pseudomonas aeruginosa* and *Vibrio cholerae*, and two strains of the plant pathogen *Xanthomonas campestris* were all detected. There was no cross-reactivity among the Gram-negative bacterial species examined in this assay, which detected about 100 cells. The assay takes less than 60 min to complete and is resistant to various media, including seawater and human serum. This strategy combines highly evolved biological materials with the optical properties of gold NPs to achieve the simple, sensitive, and specific detection of bacterial species. A schematic representation for chimeric phages and processes of pathogen detection is given in Figure 1.

**Figure 2:**
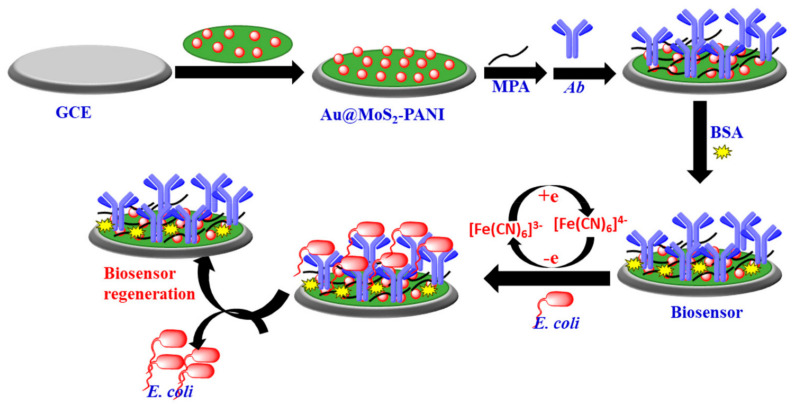
The suggested electrochemical biosensor (Au@MoS_2_–PANI nanocomposite) for *E. coli* detection; The biosensor was fabricated on the glassy carbon electrode surface and characterized by conducting the CV, DPV, and EIS techniques in 5 mM of [Fe(CN)_6_]^3-/4-^ that contained 0.1 M of KCl solution. Abbreviation: bovine serum albumin (BSA), Anti-E. coli antibody (Ab), mercaptopropionic acid (MPA), glassy carbon electrode (GCE). Reprinted from Ref. [74].

Using the Au@MoS_2_–PANI nanocomposite, Raj et al. created a label-free and extremely sensitive biosensor based on electrochemical measurement [74]. The Au@MoS2–PANI nanocomposite greatly improved the conductivity of the glassy carbon electrode, and a self-assembled monolayer of mercaptopropionic acid on the Au NPs surface was used for the covalent adsorption of antibodies to reduce non-specific bacterial pathogen binding on the electrode surface. The biosensor demonstrated great sensitivity and selectivity, with a LOD of 10 CFU mL^−1^, and identified *E. coli* in less than 30 min. In addition, the proposed biosensor showed a high linear detection range and practical use in urine samples, and electrode regeneration experiments. The suggested electrochemical biosensor for *E. coli* is shown in Figure 2.

**Figure 3:**
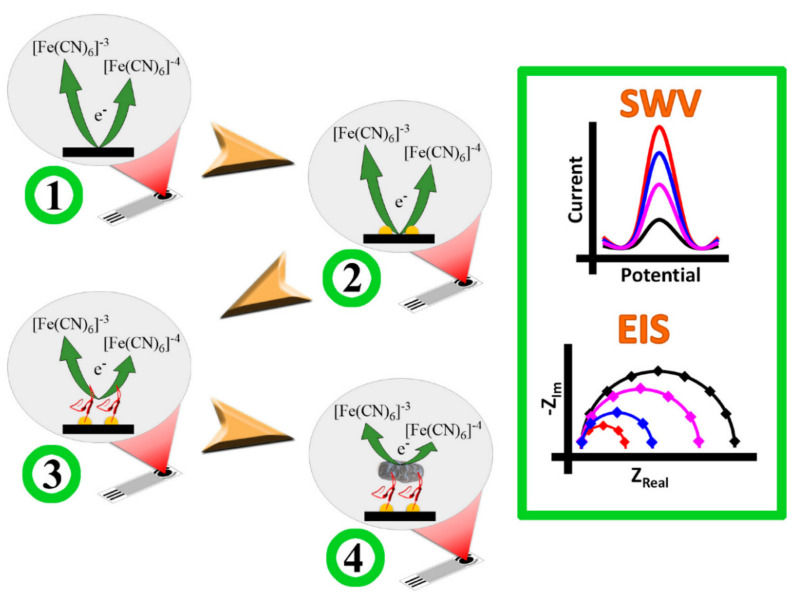
The biosensor’s construction and *E. coli* detection are depicted in this diagram. In the SWV and EIS curves, the electrochemical behaviour of the unaltered (SPE) electrode in step 1 (left) is schematically illustrated by black lines (right). SPE/ gold NPs (step 2 in left, red lines in SWV and EIS), SPE/ gold NPs /PEP (step 3 in left, blue lines in SWV and EIS), and SPE/ gold NPs /PEP/EC are the electrode’s expected responses (step 4 in left, rose lines in SWV and EIS). Reprinted from Ref. [75].

Ropero-Vega et al. described a bioinformatic architecture of a peptide based on TIR protein, an Intimin membrane protein receptor found in *E. coli* [75]. This peptide (called PEPTIR1.0) was employed as a detecting element in the biosensor (based on gold NPs modified screen-printed electrodes) for the identification of *E. coli* (Figure 3). The biosensor’s LOD and LOQ were 2 and 6 CFU mL^−1^, respectively. Furthermore, the system’s selectivity in detecting the pathogen in the presence of other bacteria, including *Staphylococcus aureus **(**S. aureus*) and *Pseudomonas aeruginosa* (*P. aeruginosa),* was statistically significant. This means this new PEPTIR-1.0-based biosensor can be used in the rapid, sensitive, and selective detection of E. coli in aqueous matrices. Pao et al. investigated carbohydrate-protein interactions and developed a label-free nanosensor to recognize *E. coli* using surface-modified Au NPs and solid-liquid contact electrification [76]. The designed TENS had a reusable potential and could identify *E. coli* in a wide range of concentrations of 2 × 10^4^–2 × 10^7^ CFU mL^−1^, with a LOD of 4 × 10^3^ CFU mL^−1^. The current work highlights the bright prospect of TENS as a new prototype of sensing technology for the label-free and rapid analysis of carbohydrate-protein interactions, as well as other pathogenic microorganisms. Figure 3 shows the biosensor’s construction and E. coli detection procedure.

#### 2.1.2. Silver NPs

Plasmonic nanosensors use the electromagnetic field localization of noble metal NPs to identify biological substances with great sensitivity. Due to their unusual optical features, nanocomposites and nanoalloys of two plasmonic metals have received much attention in recent years [96]. For example, silver-gold alloy nanohole arrays (-NHA) for the ultra-sensitive plasmonic label-free identification of *E. coli* were described by Hwang et al. [77]. Compared to monometallic gold or silver, the fully miscible silver-gold alloy had a dramatically different dielectric function in the near-infrared wavelength range. The -NHA had a significantly higher refractive index sensitivity of 387 nm RIU^−1^ than silver or silver mono-metallic nanohole arrays, which is around 40% higher. Furthermore, the -NHA provided exceptionally durable material stability against corrosion and oxidation over a one-month observation period. The ultra-sensitive -NHA enables label-free identification of *E. coli* at concentrations ranging from 10^3^ to 10^8^ CFU mL^−1^, with a LOD of 59 CFU mL^−1^. This alloy plasmonic material provides a new outlook for widely applicable biosensing and bio-medical applications.

In another study, for the construction of a new biosensor, a polymer-metal method was also used. Imran et al. developed a sensitive electrochemical nanobiosensor using positively charged chitosan stabilized silver NPs (Chi-silver NPs) for the identification of negatively charged lipopolysaccharide (LPS) or *E. coli* [78]. In the presence of both LPS and *E. coli*, glassy carbon electrodes treated with Chi-silver NPs increased its signal. Identification was achieved over a wide concentration range of 0.001–100 ng mL^−1^ and 10–10^7^ CFU mL^−1^ (many orders of magnitude). At very low concentrations, the nanosensors could reliably detect LPS and *E. coli*. Chi-AgNPs have potential as low-cost, sensitive nanobiosensors for Gram-negative bacteria, due to strong electrostatic interaction with LPS present in their outer membranes.

#### 2.1.3. QDs

As a result of their high light emission capabilities and flexibility to be modified with a range of functional groups to identify diverse analytes, QDs have recently demonstrated to be invaluable tools for biosensing applications. To take advantage of this, Wu et al. developed a new fluorescent probe based on ZnTe QDs modified with mannose (MAN) for detecting *E. coli* [79]. According to the measurements, the produced QDs had high selectivity against E. coli and good linearity in the range of 1.0 × 10^5^–1.0×10^8^ CFU mL^−1^. At pH 7.0, 25 °C, and a 20-min incubation duration, the optimal fluorescence intensity for identifying *E. coli* was observed. *E. coli* has a LOD of 4.6 × 10^4^ CFU mL^−1^ under optimal conditions. The quenching was discussed to be a static quenching procedure, which was proved by the quenching efficiency of QDs, which decreased as the temperature increased.

Zhong et al. also synthesized a zeolitic imidazolate framework-8 (ZIF-8), loaded with CdS QDs (core-shell CdS@ZIF-8) for the identification of O157:H7 and then used as a signal enhancer tag [80]. In the following, core-shell NPs were coated with polyethyleneimine (PEI) and modified with anti-*E. coli* O157:H7 antibody for specific recognition of *E. coli*. The detection range of the nanosensor was 10–10^8^ CFU mL^−1^ and LOD of 3 CFU mL^−1^ (Signal/noise = 3). Interestingly, the sensitivity of CdS@ZIF-8 for the detection of *E. coli* was 16 times greater than CdS QDs.

**Figure 4:**
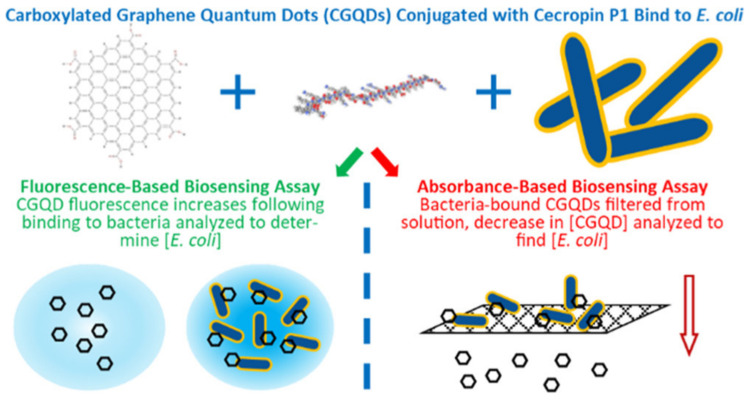
Fluorescent-assisted nanosensor array based on graphene QDs attached cecropin P1 for identification of pathogens. Reprinted from Ref. [81].

Bruce et al. used a biosensing test that emphasized monitoring changes in fluorescence intensity to examine the application of conjugated carboxylated graphene CGQDs to identify *E. coli* [81]. Cecropin P1, a naturally occurring antibacterial peptide that aids in the adhesion of CGQDs to *E. coli* cells, was conjugated to CGQDs. The findings could be useful in instances where rapid, consistent detection of bacteria in liquids, such as drinking water, is required, especially considering the low range of *E. coli* concentrations (10^3^ to 10^6^ CFU mL^−1^) within which two biosensing tests have been proven to perform together. These findings have the potential to be used in situations where rapid, reliable detection of bacteria in liquids, such as drinking water, is required. A schematic representation for a fluorescent-assisted nanosensor array, based on graphene QDs attached cecropin P1 for identifying pathogens is given in Figure 4.

#### 2.1.4. Carbon Nanomaterials

Carbon nanomaterials have been used to make electrochemical biosensors in recent years due to their unique mix of intrinsic features, such as high conductance, durability, and bioactivity, making them a suitable candidate for bio-sensing material [82]. For example, by polymerizing aniline (PANI) in the presence of carbon dot and zinc oxide nanorod, a conductive nanocomposite electrode, CDs/ZnO/PANI, was successfully produced for the identification of *E. coli* [97]. The electrical conductivity of CD/ZnO/PANI was shown to boost *E. coli* detection capability. The proposed electrochemical biosensor showed good selectivity, detecting *E. coli* O157:H7 in water samples with LOD of 1.3 × 10^18^ M.

**Figure 5:**
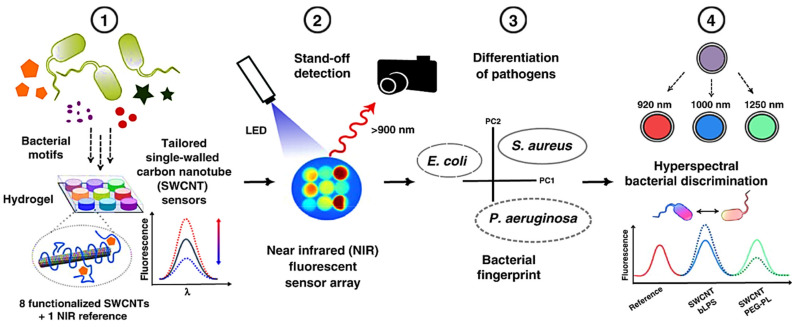
A schematic diagram for identification of pathogens. (**1**) SWCNTs-based nanosensor in the presence of pathogens produce a specific fluorescence signal, (**2**) PEG based-hydrogel with 8 fluorescent sensors remotely observed in NIR, (**3**) Growth of pathogens on hydrogel sensor change sensor array fingerprint and can discriminate different infections, (**4**) Multimodal nanosensors can specify for detection and discriminate different pathogens. Reprinted from Ref. [83].

Nibler et al. developed a set of near-infrared (NIR) fluorescence nanosensors based on single-walled carbon nanotubes (SWCNTs) and utilized them for remote fingerprinting of clinically important bacteria [83]. Based on their metabolic fingerprint, this multiplexed sensor array was able to identify the presence of bacteria and differentiate the majority on a species level. Even closely related significant pathogens (*S. aureus* and *Staphylococcus epidermidis*) isolated from various human illnesses may be differentiated. This type of multiplexing with NIR fluorescent nanosensors enables remote detection and differentiation of important pathogens and the potential for smart surfaces. A schematic diagram for identifying pathogens by this method is given in Figure 5, showing that a SWCNTs-based nanosensor, in the presence of pathogens, produces a specific fluorescence signal and a PEG based-hydrogel, with 8 fluorescent sensors remotely observed in NIR. Additionally, the growth of pathogens on the hydrogel sensor changes the sensor array fingerprint and can discriminate different infections.

Kaur et al. reported the invention of a label-free impedimetric aptasensor for specific and accurate identification of *E. coli* O157:H7, using a nanostructured platform made of boron-carbon nanorods coated with nickel NPs (BC-Ni) [84]. The electrochemical aptasensor was shown to identify *E. coli* O157:H7 preferentially in water, juice, and faeces samples with a LOD of 10 CFU mL^−1^ and a dynamic detection range of 100 to 10^5^ CFU mL^−^^1^.

Lee et al. developed a continuous flow multijunction biosensor for detecting *E. coli* K12 and *S. aureus* at the same time [80]. Gold-plated tungsten wires coated with polyethyleneimine, and single-walled carbon nanotubes were used to make junction biosensors. Streptavidin and biotinylated antibodies specific to *E. coli* K12 and *S. aureus* were used to functionalize each junction. Compared to the stationary sensor, the constructed junction sensor linked with the fluidic channel showed improved electric signal outputs for identifying *E. coli* K12. In the sensing range of 10^2^–10^5^ CFU mL^−1^, a linear regression was seen for both the *E. coli* and *S. aureus* functionalized array sensors. Within 2 min, multiplexed identification of bacteria at sensing levels as low as 10^2^ CFU mL^−1^ was obtained for *E. coli* K12 and *S. aureus*.

#### 2.1.5. Metal-Organic Frameworks (MOFs)

Metal-organic frameworks (MOFs) are chemical types that combine metal ions or clusters with organic ligands to produce one, two, or three-dimensional architectures. They are a type of coordination polymer that has the unique property of being porous. MOFs are considered a suitable medium for the construction of biosensors for various analytes found in the environment [85]. In this light, Gupta et al. reported employing a water-dispersible terbium MOF to identify *E. coli* (Tb-BTC; BTC, 1,3,5-benzenetricarboxylic acid) [98]. Tb-BTC was bio-interfaced with anti-*E. coli* antibodies before being tested as an *E. coli* biosensor. The biosensor can identify analytes with concentrations ranging from 1.3 × 10^2^ to 1.3 × 10^8^ CFU mL^−^^1^, with a detection limit of 3 CFU mL^−1^, and has a detection time of 5 min and a total analysis time of 20–25 min.

The design of a Cu-MOF based electrochemical biosensor for the very selective detection of *E. coli* bacteria was reported by Gupta et al. [86]. Cu_3_(BTC)_2_ (BTC = 1,3,5-benzenetricarboxylic acid) was combined with polyaniline (PANI) to create a MOF-based electrochemically active platform. Cu_3_(BTC)_2_-PANI thin films were bio-interfaced with anti-*E. coli* antibodies and used as a biosensing electrode on an indium-tin-oxide (ITO) substrate. The sensor described above, which used the electrochemical impedance spectroscopy (EIS) signal measuring methodology, had great sensitivity for detecting very low *E. coli* (2 CFU mL^−1^) concentrations in a fast reaction time (2 min) and was also sensitive in the presence of other non-specific bacteria. This new MOF/PANI based detection platform for E. coli has shown improved performance compared to many of the previously reported electrochemical biosensors.

#### 2.1.6. Silica NPs

To examine and unravel biological processes and related mechanisms, accurate, low-toxicity, and real-time biochemical tests are necessary. Sensors and probes made of silicon nanomaterials have the ability to meet the above-mentioned criteria [99]. To test this feature, Jenie et al. described the production of fluorescent silica NPs (SNP-RB) from natural amorphous silica and evaluated their efficacy as an *E. coli* biosensor [87]. The presence of Rhodamine B in the silica matrix was confirmed by Fourier Infrared (FTIR). The SNP-RB exhibited an irregular structure architecture with a particle diameter of approximately 20–30 nm, according to TEM evaluation. The highest fluorescence spectrum of SNP-RB was obtained at 580 nm, which was then used to test the fluorescent NPs’ detection accuracy against *E. coli*. The detecting approach was based on SNP-fluorescence-quenching RB’s mechanism, which gave a linear *E. coli* concentration range of 10–10^5^ CFU mL^−1^ with a LOD of 8 CFU mL^−1^. After only 15 min of incubation with *E. coli*, SNP-RB showed a quick response time. The biosensor’s specificity was tested, revealing that the SNP-RB only gave a quenching response to live *E. coli* bacteria. When compared to traditional 3-day bacterial experiments (such as ELISA, PCR and culturing method), the adoption of SNP-RB as a sensing platform lowered response time dramatically, while also providing good analytical performance in terms of selectivity and sensitivity.

**Figure 6:**
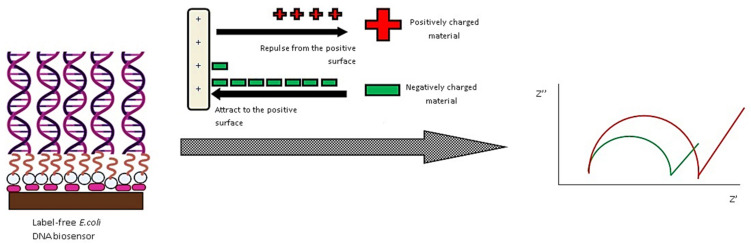
Schematic procedure for label free detection of *E. coli* by DNA attached-hollow silica NPs. Reprinted from Ref. [88].

Yuhana et al. developed a new label-free electrochemical DNA biosensor for detecting *E. coli* bacteria in environmental water samples. The aminated DNA probe was mounted on 3-aminopropyltriethoxysilane-functionalized hollow silica microspheres (HSMs) and placed onto a screen-printed electrode (SPE) carbon paste with integrated gold NPs [88]. The biosensor’s selectivity and specificity were improved. Without a redox mediator, the label-free *E. coli* DNA sensor had a dynamic linear response range of 1 × 10^−10^ M to 1 × 10^−5^ M (R^2^ = 0.982), with a LOD of 1.95 × 10^−15^ M. The designed DNA biosensor had a sensibility that was equivalent to non-complementary and single-base mismatched DNA. At 4 °C and pH 7, the DNA biosensor showed a steady response after 21 days of storage. Over three regeneration and rehybridization cycles, the DNA biosensor response was regenerated. The schematic procedure for label-free detection of E. coli by DNA attached-hollow silica NPs was depicted in Figure 6.

#### 2.1.7. Magnetic NPs

**Figure 7:**
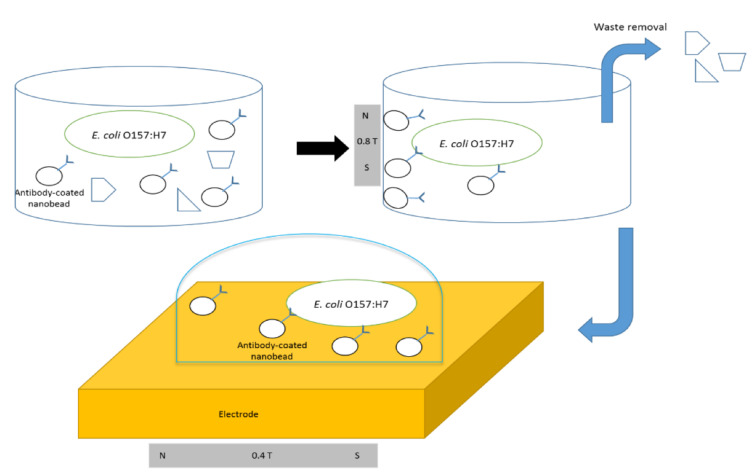
Architecture illustration of antibody-covered nanobeads and low concentration detect of *E. coli* using a magnetic field procedure. Reprinted from Ref. [89].

Magnetic NPs have been widely utilized in biosensing for pathogen identification, mainly for the immunomagnetic separation of bacteria from a sample, or as labels to improve the biosensor’s responsiveness [90,100]. Wang et al. developed an impedance immunosensor based on magnetic nanobeads and screen-printed interdigitated electrodes for the fast detection of *E. coli* O157:H7 [89]. Anti-*E. coli* antibody-coated magnetic nanobeads were combined with an *E. coli* sample and used to extract and concentrate the bacteria. The substance was immersed in a redox probe solution and placed on an interdigitated electrode that had been screen-printed. The impedance was examined after a magnetic field concentrated the cells on the electrode’s surface. Without pre-enrichment, the impedance immunosensor could identify *E. coli* O157:H7 at a concentration of 1400 bacterial cells in a volume of 25 L in less than 1 h. Between 10^4^ and 10^7^ CFU mL^−1^, a linear relationship between bacteria concentration and impedance value was observed. Despite the presence of a redox probe during impedance measurement, analysis of the equivalent circuit model revealed that the impedance shift was predominantly caused by two factors: double-layer capacitance and resistance owing to electrode surface roughness. The magnetic field and impedance were simulated using COMSOL Multiphysics software. The architecture illustration of antibody-covered nanobeads and the low concentration detection of *E. coli* using a magnetic field procedure is given in Figure 7.

Varshney et al. developed and tested an impedance biosensor based on an interdigitated array microelectrode (IDAM) combined with magnetic NP–antibody conjugates (MNAC) for the quick and precise identification of *E. coli* O157:H7 in ground beef specimens [90]. Biotin-labelled polyclonal goat anti-*E. coli* antibodies were immobilized onto streptavidin-coated magnetic NPs, which were utilized to extract and concentrate *E. coli* O157:H7 from ground beef samples. In the presence of 0.1 M mannitol solution, the magnitude of impedance and phase angle were evaluated over a frequency range of 10 Hz–1 MHz. The biosensor’s lowest LOD for *E. coli* O157:H7 in pure culture and ground beef samples were 7.4 × 10^4^ and 8.0 × 10^5^ CFU mL^−1^, respectively. By concentrating bacterial cells linked to MNAC in the active layer of IDAM above the surface of electrodes with the help of a magnetic field, the sensitivities of the impedance biosensor were increased by 35%. According to equivalent circuit analysis, the impedance shift caused by the presence of *E. coli* O157:H7 on the surface of IDAM was caused by bulk resistance and double-layer capacitance. This impedance biosensor did not use surface immobilization methods, redox probes, or sample incubation. From sampling to testing, the entire detection time was 35 min.

In an interesting paper, Lee et al. combined two features of localized surface plasmon resonance and target separation of immunomagnetic NPs for the low-concentration detection of O157:H7 in lettuce [91]. Immunomagnetic NPs were synthesized based on core Fe_3_O_4_ (10 nm) and shell of Au. Then, core-shell multifunctional immunomagnetic NPs was modified with anti-*E. coli* O157:H7 antibodies for simultaneous identification and isolation of O157:H7. The nanosensor could detect bacteria in the lettuce matrix with a LOD of 1 log CFU mL^−1^ and no-enrichment procedure. The method, which requires no pre-enrichment, provides an alternative to conventional microbiological detection methods and can be used as a rapid screening tool for many food samples.

#### 2.1.8. ZnO NPs

**Figure 8:**
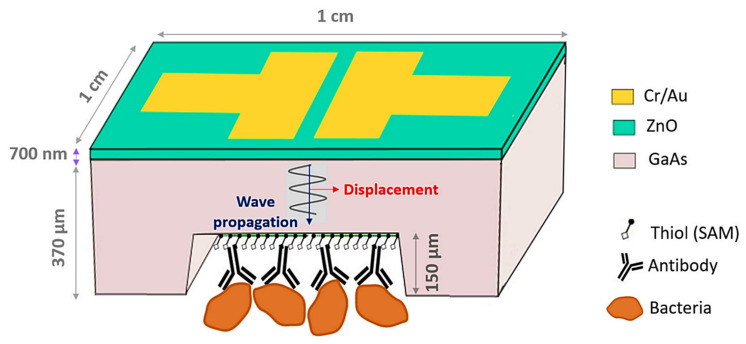
Schematic construction of ZnO/GaAs BAW nanosensor for identification of *E. coli.* Reprinted from Ref. [101].

Pathogenic bacteria must be detected in various disciplines, including food safety, environmental water analysis, and clinical diagnostics. Despite the fact that fast and specific procedures based on the quick and simple binding of recognition elements and targets have been developed, the sensitive identification of bacterial pathogens has been limited due to their low targets. As ZnO NPs have a large binding capacity, they can provide additional reactive sites to bind with bacterial targets, improving detection sensitivity significantly [92]. Chawich et al. created a regenerable bulk acoustic wave (BAW) biosensor for the quick, label-free, and specific detection of *E. coli* in a liquid medium [101]. The biosensor’s architecture consists of a GaAs membrane with a thin piezoelectric ZnO layer on its top surface. BAWs can be generated by lateral field stimulation using a pair of electrodes placed on the ZnO layer. Alkanethiol self-assembled monolayers and antibodies against *E. coli* were fictionalized on the membrane’s back surface. The immobilization of antibodies was studied as a function of antibody suspension concentration, pH, and incubation period in order to maximize bacterial immunocapture. Detection tests in various conditions for bacterial suspensions ranging from 10^3–^10^8^ CFU mL^−1^ were used to assess the biosensor’s performance. For suspensions ranging from 10^3–^10^7^ CFU mL^−1^, a linear relationship between the frequency response and the logarithm of *E. coli* concentration was observed, with the biosensor’s LOD estimated to be 10^3^ CFU mL^−1^. The biosensor capability for the desired operation in complicated biological media is demonstrated by the 5-fold regeneration and high selectivity towards *E. coli* identified at 10^4^ CFU mL^−1^ in a suspension coloured with *Bacillus subtilis* at 10^6^ CFU mL^−1^. The schematic construction of a ZnO/GaAs BAW nanosensor for identification of *E. coli* is depicted in Figure 8.

**Figure 9:**
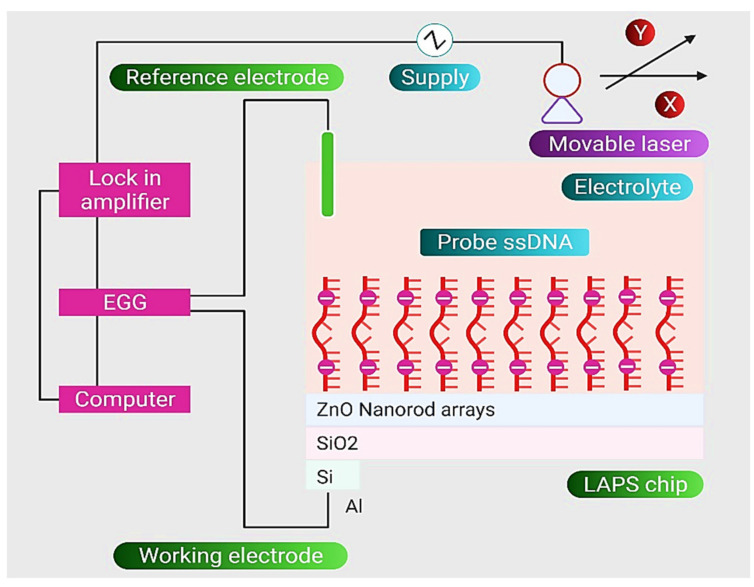
Schematic diagram of the nanosensor arrangement, built up from ZnO nanorods arrays, probe ssDNA, and silica layer for detection of *E. coli*. Reprinted from Ref. [93].

Tian et al. developed a label-free nanosensor based on the light-addressable potentiometric sensor (LAPS) and ssDNA-ZnO nanorod arrays (NRAs) [93]. The recognition element for specific detection of *E. coli* O157:H7 DNA was constructed by attachment of ssDNA on the surface of LAPS. The developed nanosensor was added to a solution of *E. coli* ssDNA molecules until the hybridization procedure of target DNA and probe was completed. The findings show that different signal variations can be observed and recorded in order to identify *E. coli* ssDNA. The target ssDNA had a LOD of 1.0 × 10^2^ CFU mL^−1^ for *E. coli* O157:H7 detection in solution. All the results demonstrate that this DNA biosensor, based on the electrostatic detection of ssDNA, provides a novel approach for the sensitive and effective detection of bacterial DNA, which has promising prospects and potential applications in the quality control of food and water. A schematic diagram of the nanosensor arrangement built up from ZnO nanorods arrays, probe ssDNA, and silica layer for detecting E. coli is shown in Figure 9.

## 3. Nanomaterials for Treatment of *E. coli* Infections

**Figure 10:**
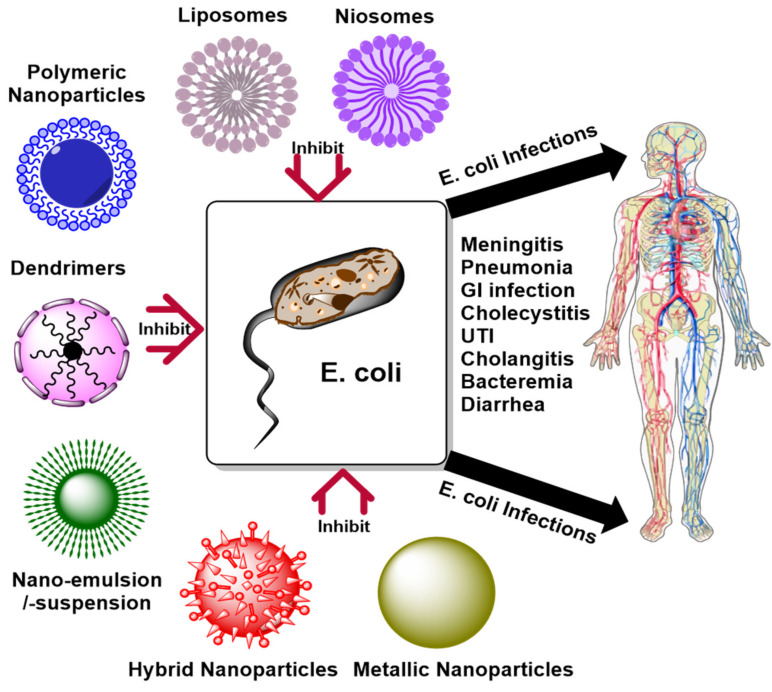
Different types of nanomaterials used to inhibit *E. coli*.

In the 21st century, NPs have emerged as the most innovative system for drug delivery and therapeutic purposes. Different nanomaterials were used to prepare NPs according to their unique characteristics, such as controlled drug release, site-specific targeting, lower side-effects and toxicities, and higher therapeutic efficiency. Meanwhile, bacterial infections are suitably treated with these NPs with promising outcomes. Figure 10 shows different types of NPs which are utilized for the drug delivery and treatment of *E. coli* infection.

### 3.1. Polymeric Nanocarriers

Polymeric nanocarriers have a leading biomedical role in drug delivery and therapeutics. Various polymeric NPs were devised to target *E. coli* bacteria and to retard bacterial “planktonic” and “biofilm” growth [102]. Polymeric nanocarriers enable the controlled release of antibiotic drugs over a longer time, larger blood circulation time, and the ability to penetrate physiological barriers to reach the target infected tissue [102,103]. Several natural (chitosan, albumin, gelatin, etc.) and synthetic materials (poly lactic-*co*-glycolic acid (PLGA), polylactic acid (PLA), polyethyleneimine (PEI), polycaprolactone (PCL), etc.) are utilized to construct polymeric nanocarriers [103].

In a recent attempt, nettle essential oil was encapsulated inside chitosan NPs to assess its antibacterial activity [103]. The nanoformulation was prepared through the emulsion-ionic gelation method. Nettle oil encapsulation within the chitosan NPs improves its solubility and controls its rapid volatility. The formed NPs had a good oil retention rate, which conformed to FTIR and XRD data. Therefore, the antioxidant and antibacterial effects of the nettle oil-loaded chitosan NPs were promising, thus paving the way for its use as a future non-toxic therapy. The DPPH free radical scavenging assay revealed higher antioxidant activity in the encapsulated oil than the free oil. Similarly, in-vitro antibacterial assay showed that encapsulated oil has increased inhibition halo (3.95 cm) against *E.coli* [103]. 

Similarly, cranberry proanthocyanidin chitosan nanocomposites were prepared without using any cross-linker. Instead, ratio adjustment of the proanthocyanin and chitosan modulate hydrogen binding were used [104]. The nanoformulation has shown extensive agglutination action against extra-intestinal *E. coli* bacteria and retarded invasion of gut epithelial cells by the *E. coli* in a dose-dependent manner [104].

Further, the intrinsic anti-bacterial property of chitosan was enhanced through its mannosylation via reductive amination reaction, and NPs were prepared through ionic gelation [105]. Mannose-modified chitosan NPs demonstrated specific interaction with resistant *E. coli* bacterial membranes and improved antibiofilm properties as compared to plain chitosan NPs. The modified chitosan NPs are of special interest in combating *E. coli* associated multidrug resistance. The anti-bacterial activity was assessed through antibiofilm assay, time-kill activity, and polystyrene adherence. Findings indicated greater inhibition and biofilm disruption of *E. coli* and other gram-positive bacteria, thus emphasizing its role against multidrug-resistant, biofilm-forming bacteria in resistant infections. [105].

Apart from natural polymers, charged synthetic polymers were also used against bacterial infections. In one such attempt, a photosensitizer, Chlorin e6 (Ce6) was encapsulated inside charge-conversion polymeric (poly(PEG-*co*-β-amino ester) nanocarriers for targeting *E. coli* bacteria in urinary tract infections [106]. The Ce6 was released from the nanocarrier at the targeted infection site, and Ce6, under photodynamic therapy, generated reactive oxygen species to kill the bacteria. Surface charge conversion of the polymeric nanocarriers in the weakly acidic environment of urinary tract infection additionally facilitated recognition and bacterial interaction. Ce6-loaded polymeric nanocarriers exhibited higher antibacterial activity against *E. coli* and efficiently treated a mouse cystitis model under photodynamic therapy [106].

Further, dual antibacterial and antiparasitic multifunctional PLGA NPs were synthesized with coloaded caffeic acid phenethyl and juglone drugs [107]. The combined rationale was to combat bacterial drug resistance and to achieve controlled release of the drug. The combined synergistic actions of the drugs inside the PLGA NPs were profoundly greater to inhibit *E. coli* (MIC: 12.5 μg/mL^)^ and *Leishmania* (lower IC50 value), proving the efficacy of the nanoformulation [107].

Another innovation is developing a novel antibacterial polymer, cationic acrylate copolymeric polyvidone with double active centres and then complexed with iodine [108]. Further, their NPs were synthesized, which exhibited enhanced anti-bacterial activity against *E. coli* and *S. aureus*. The formed NPs endured antibacterial properties to the dyes, inks, and different coatings. It was found that the NPs have long-term anti-bacterial properties with 99% efficacy at a concentration of 40 µg/mL [108].

PCL is another polymer with wide applications in nanomedicine. In a study, chlorhexidine-loaded PCL nanospheres were prepared and coated on the urinary catheters for anti-bacterial action against uropathogens, including *E. coli*. The total amount of chlorhexidine loaded on the coated catheter was around 4.55 mg. The NP-coated catheter was immersed in artificial urine containing microorganisms. Drug release was exceeded up to 2 weeks, with satisfactory inhibition of bacterial growth and proliferation into the catheter up to 14 days [109]. This innovation aided the urinary catheter with long-term anti-bacterial action and protected it in the urinary environment.

### 3.2. Lipidic Nanocarriers

Lipids are a basic component of the cell membrane, hence, lipid-based nanovesicles mimic natural components and easily transfer the drug molecule inside the cell. This property allows lipid-based nanovesicles to specifically target bacterial cells.

Nanostructured lipidic carriers (NLCs) are lipidic carriers made up of lipids, surfactants, and co-surfactants, with a higher capacity to load drugs. For instance, ceftriaxone-loaded NLCs were prepared using the double emulsion solvent evaporation method for bactericidal action against *E. coli* [110]. Ceftriaxone has a bactericidal action; however, it is linked with several side effects. Therefore, ceftriaxone loaded NLCs were prepared to accommodate a low dose of the drug, while maintaining its anti-bacterial activity. For the said purpose, Haftyzer-Van Krevelen’s method was employed to adjust the ratios of NLCs components to produce optimal sized NLCs. It was noticed that the prepared ceftriaxone-loaded NLCs could kill *E. coli* even in a dose that was half of the free drug dose [110].

Solid lipidic nanocarriers (SLNs) are another versatile lipidic nanocarrier with long-term stability. A novel class of SLNs was developed using propylene glycol monopalmitate and glyceryl monostearate lipidic mixtures. Carvacrol was loaded as a drug with >98% encapsulation efficiency. Carvacrol-loaded SLNs demonstrated higher anti-bacterial effects against *E. coli* O157:H7 strains, thus proving their role in delivering lipophilic drugs for biomedical applications and prospective role in treating *E. coli* infections [111].

Liposomes are a bi-layered vesicular system with biocompatible, biodegradable, and non-toxic nature, and can encapsulate all types of drugs from hydrophilic to hydrophobic. In a recent attempt, endolysin BSP16Lys containing cationic DPPC liposomes were prepared to act against antibiotic-resistant gram-negative bacteria [112]. Endolysins can degrade bacterial cell wall peptidoglycan but cannot penetrate the outer membrane of gram-negative bacteria. Therefore, cationic liposomes can deliver these endolysins safely across the outer membranes of *E. coli*. BSP16Lys-liposome have reduced about 1.6-log colony-forming units (CFU)/mL of the viable *E. coli*, demonstrating the potential of these liposomes against resistant *E. coli* infections [112]. Likewise, several other lipidic structures are efficiently combating *E. coli* led infections. Furthermore, antimicrobial lipid, dioctadecyldimethyammonium bromide (DODAB), was used to form lipidic bi-layered systems and entrap antimicrobial peptide, gramicidin [113]. Gramidine loaded DODAB lipidic formulations have broadened the anti-bacterial spectrum and efficiently killed *E. coli* with no toxicity against eukaryotic cells [113]. 

For enhanced coverage against bacterial infections, a combination of different types of nanocarriers or materials is employed. For instance, ceftriaxone-loaded polymer-lipid hybrid NPs were prepared using the self-assembly method [114]. Chitosan was used as a polymer and glycerol monostearate as a lipidic material. The hybrid polymer-lipid NPs have sustained release characteristics and an effective mortality rate against *E. coli* [114].

Similarly, chitosan-coated nanostructured lipidic carriers (NLCs) were developed to combat *E. coli* infection and biofilm formation on catheters [115]. *E. coli* biofilms were grown on the catheters and treated with chitosan-NLCs. The biofilms were collected after 24 h of treatment with chitosan-NLCs. Results indicated that the chitosan-NLCs hybrid system considerably inhibited the viability of biofilms at all ages and could control the growth of both young and mature *E. coli* biofilms on the catheters [115].

### 3.3. Metallic Nanocarriers

Different metallic nanocarriers, such as iron (Fe), silver (Ag), gold, zinc (Zn), etc., have shown promising roles as antibacterial and antibiotic drug delivery carriers.

Iron oxide NPs are of particular importance in metallic NPs. For instance, iron oxide NPs with biocompatible oleic acid coating were synthesized in the size of 10.64 nm to combat *E. coli* infection [116]. The synergistic effect of both iron oxide NPs and the encapsulated antibiotic drug produced increase H^+^ conductance with lower flux with detrimental antibiotic action against antibiotic-resistant strains of *E. coli*, i.e., ampicillin-resistant *E. coli* DH5α-Puc 18 and kanamycin-resistant *E. coli* Parg-25. Thus, iron-oxide-oleic acid NPs have the potential to be used as a strong anti-bacterial agent against antibiotic-resistant organisms in the future [116].

Magnetite is one of the forms of iron oxide. Magnetite NPs were prepared by the green, surfactant-free electrochemical method [56]. In this study, the sole benefits of nano-magnetite had been investigated with no other agent or drug. Nano-magnetite had shown potent antibacterial action against *E. coli* with a minimum inhibitory concentration (MIC) of 2.8 µg/mL. The MIC is about 100 times lesser than the human toxic, confirming the low toxicity potential. The only dose above 100 µg/mL for the 2 weeks produced damage to the kidney and the liver. Thus, it demonstrated that nano-magnetite itself could be used to treat infections with a wide therapeutic window [56].

Zinc is an important micro-ingredient for normal body function with natural anti-bacterial activity. In a study, zinc oxide (ZnO) NPs were synthesized through green, eco-friendly, and economical methods using 4 plant extracts: *Beta vulgaris, Brassica oleracea, Cinnamomum verum*, and *Cinnamomum tamala*. In-vitro characterization demonstrated suitable size and morphology. Moreover, ZnO NPs proved their anti-bacterial activity against *E. coli* and *S. aureus* [51].

Ag is a well-known antimicrobial agent. In the recent past, various studies reported the efficacy of Ag NPs [117,118]. In one of these studies, a Ag NPs led anti-bacterial mechanism was investigated [117]. Prepared Ag NPs interacted with *E. coli* and demonstrated a 100% inhibition ratio with two stepped inhibition and sterilization process against *E. coli*. The whole inhibition-sterilization process was analysed through growth curves, FTIR, SEM, and MDA concentrations [117].

Magnetic nanocomposites consisting of BaFe_12_O_19_/xCoFe_2_O_4_ were fabricated using the sol–gel method [119]. In-vitro characterization techniques such as scanning electron microscopy (SEM), X-ray diffractometer (XRD), and vibrating magnetometer (VSM) confirmed magnetic and structural properties. Anti-bacterial activity against several fungal and bacterial strains, including *E. coli* was found promising and thus, proving their efficacy as an antimicrobial agent [119].

### 3.4. Other Nanocarriers

Niosomes are a vesicular system prepared from non-ionic surfactant and a stabilizer. Due to their peculiar nature, niosomes have a wide window to encapsulate various types of drug moieties. In a recent study, simvastatin-loaded niosomal gel was prepared for the activity against *E. coli* and *S. aureus*. Suitable-sized niosomes are formed from appropriate ratios of span 80, drug, and cholesterol. Prepared niosomes have good stability and good antibacterial activity [120].

Nanoemulsion is a versatile liquid system containing two immiscible liquids with the help of an emulsifier. Liquid dosage forms have importance in killing microorganisms. In an effort, ε-polylysine and D-limonene nanoemulsion was formulated using Tween-80 as a surfactant. Both components have shown greater synergistic effect against bacteria, including *E. coli*, *S. aureus*, *Bacillus subtilis*, etc. [121]. 

Likewise, thyme oil and sodium caseinate-based neutral self-assembled nanoemulsion was prepared for antimicrobial action [122]. Encapsulated thyme oil exhibited 28 times more efficacy in combating *E. coli* O157:H7 and *S. aureus* in the ***tryptic soy broth*** and milk than free thyme oil. Thus, the nanoemulsion can be used as a preservative and treat infections [122].

**Figure 11:**
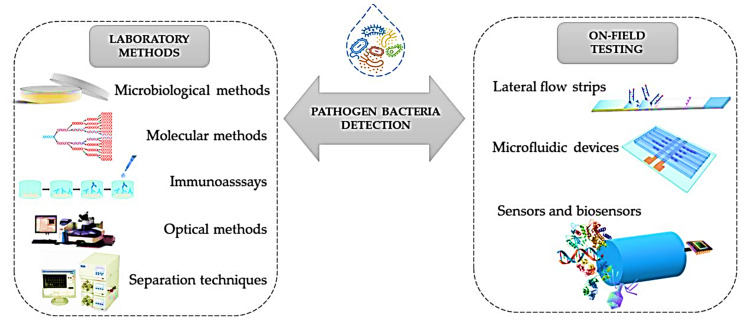
Schematic representation of the current analytical methods and POC devices applied to detect *E. coli* Reprinted from ref. [125].

Dendrimers are highly branched, symmetric, nanosized molecules used for drug delivery, diagnostics, and therapeutic purposes. Researchers developed bioinspired peptide Trp-terminated dendrimers as an effective treatment of *E. coli* infection. Trp-terminated dendrimers have demonstrated splendid activity against extended-spectrum beta-lactamases (ESBL)-producing and multidrug resistance (MDR) isolates of *E. coli*. The peptide-based dendrimers were found to be stable in plasma over a longer period with minimal hemolysis and low genotoxicity [123]. A summary of different types of nanocarriers for the *E. coli* inhibition is compiled in Table 2 and illustrated in Figure 11.

## 4. Point of Care (POC) Devices for Clinical Applications

Pathogens, and all diseases associated with them, are a significant concern worldwide [125]. Diagnostic tests have been suggested to prolong the effectiveness of current antimicrobials; culture and other conventional diagnostics are hindered in their practicality as they are time- and labour-intensive to perform. POC testing is performed near where the patient is being treated and can provide timely results that allow evidence-based clinical interventions to be made (Figure 11) [126]. For example, a portable multiplexed bar-chart SpinChip (MB-SpinChip) integrated with NP-mediated magnetic aptasensors was developed for visual, quantitative instrument-free detection of multiple pathogens. This versatile multiplexed SpinChip combines aptamer-specific recognition and NP-catalysed pressure amplification to achieve a sample-to-answer output for sensitive point-of-care testing (POCT). This user-friendly MB-SpinChip allows visual, quantitative detection of multiple pathogens simultaneously with high sensitivity, but without utilizing any specialized instruments. Using this MB-SpinChip, three major foodborne pathogens, including *Salmonella enterica, Escherichia coli*, and *Listeria monocytogenes,* were specifically quantified in apple juice with limits of detection of about 10 CFU/mL [127]. In another study, a smartphone-based nanosensor was developed to detect zika virus (ZIKV) infection. In this light, a nanomotor-based bead-motion cell phone (NBC) system was developed for the immunological detection of ZIKV. The presence of a virus in a testing sample results in the accumulation of platinum (Pt)-nanomotors on the surface of beads, causing their motion in H_2_O_2_ solution. Then, the virus concentration is detected in correlation with the change in beads motion. The developed NBC system could detect ZIKV in samples with virus concentrations as low as 1 particle/μL. The NBC platform technology has the potential to be used in the development of point-of-care diagnostics for pathogen detection and disease management in developed and developing countries [128]. Of course, new simulation and machine learning approaches can help better optimize these devices [129,130,131]. The schematic representation of the current analytical methods and POC devices applied for the detection of E. coli are shown in Figure 11.

## 5. Regulatory Landscape of Nanotechnology in Biomedical Applications

The safety assessment of medical devices containing or deriving from nanotechnology is carried out by the US-FDA’s Centre for Devices and Radiological Health (CDRH), housing a Nanotechnology Regulatory Science Research Programme that is based on three pillars: physicochemical characterization methods, in vitro and in vivo models, and (toxicological) risk assessment [132,133]. The types of devices that incorporate nanotechnology include antimicrobial, dental, orthopaedic, neurological, and combination devices and in vitro diagnostic tools. They use various nanomaterials, including silver, zirconia, titanium and titanium dioxide, iron oxides, polymers, gold, graphene etc. Safety assessment of such medical devices should encompass the determination of the rate and magnitude of the nanomaterials into the body for which fit-for-the-purpose in vitro tests would be desirable [134]. Moreover, advanced toxicological risk assessment approaches should support the understanding that the release and patient exposure results in adverse health impacts. It is important to know whether NPs affect the accuracy and/or reliability of standard biocompatibility or toxicity test assays, such as cytotoxicity and genotoxicity. Because of the vast number of sizes, shapes, and chemistry of nanomaterials, there is the need for the development of in vitro models (2D, 3D, organ on a chip, organoids) and in silico models in order to predict human responses and improve in vitro to in vivo extrapolations [133].

## 6. Conclusions

NPs have been shown to have considerable potential in the development of bacterial sensing devices. Bacterial sensing can be conducted in a lab setting by evaluating physiological fluids, or in vivo by tracking bacteria in real-time within the body. In a label-free methodology, NPs can act as both a detection element and a reporter when the surface is coupled with a specific recognition element. Integrating artificial intelligence, a sub-type of machine learning with nanotechnology, is paving the way for advancements in microbe detection systems and is working closely toward the human dream of having a sensor that fully fits all of the aforementioned criteria. Even though several biosensors for detecting food-borne diseases have been created, researchers continue to face difficulties in fabricating biosensors for the accurate and reliable assessment of microorganisms in real food samples. In short, a variety of nanocarriers have shown promising efficiency in delivering antibiotic to the *E. coli* infected area and inhibiting *E. coli* at a much lower dose. Moreover, some of the nanomaterials have antibacterial action and produced synergistic action along with antibiotics to combat *E. coli*.

## Figures and Tables

**Table 1:** Summary of different nanomaterials in the detection of *E. coli*.

Nanostructure	Type	Key Feature	Ref.
Au NPs	Labeled gold NPs	A perfect Detect of *E. coli* 500 CFU mL^−1^ in 1 mL of sample	[71]
	DNA-gold NPs	Detection of bacteria with low concentration of 2 × 10^3^ CFU mL^−1^	[72]
	Protein-gold NPs	No cross-reactivity for Gram-negative pathogens	[73]
	gold@MoS₂–PANI nanocomposite	10 CFU mL^−1^ for LOD in just 30 min	[74]
	Peptide-gold NPs	LOD and LOQ for *E. coli* measurement was 2 and 6 CFU mL^−1^, respectively	[75]
	Protein-gold NPs	A perfect LOD of 4 × 10^3^ CFU mL^−1^, reusable potential, and wide-range analysis of 2 × 10^4^–2 × 10^7^ CFU mL^−1^ for *E. coli* detection	[76]
Ag NPs	Ag-gold alloy nanohole arrays	Label-free detection, wide-range analysis of 10^3^–10^8^ CFU mL^−1^ and LOD of 59 CFU mL^−1^	[77]
	Polymer-Ag NPs	Wide range analysis of 0.001–100 ng mL^−1^ and 10–10^7^ CFU mL^−1^	[78]
QDs	Mannose-ZnTe QDs	Good selectivity and a perfect linearity range of 1.0 × 10^5^–1.0 × 10^8^ CFU mL^−1^ toward *E. coli*	[79]
	CdS QDs@MOF	Suitable linear range of 10–10^8^ CFU mL^−1^, LOD of 3 CFU mL^−1^ and S/N of 3	[80]
	carboxylated graphene QD	Detection of pathogen in drinking water in low concentrations of *E. coli* (10^3^–10^6^ CFU mL^−1^)	[81]
Carbon nanomaterials	Carbon dot/ZnO/PANI	Perfect selectivity and good LOD of 1.3 × 10^−18^ M *E. coli* in water	[82]
	SWCNTs	Detect the presence of specific bacteria based on metabolic fingerprint and differentiate among other pathogens	[83]
	Aptamer-BC-Ni NPs	Selective detection of *E. coli* with a LOD of 10 CFU mL^−1^ and wide range detection of 100–10^5^ CFU mL^−1^ in juice, water, and fecal	[84]
	POE-SWCNTs	Multiplexed detection and a LOD of 10^2^ CFU mL^−1^ within 2 min	[80]
MOF	Tb-BTC	Wide Detection range of 1.3 × 10^2^–1.3 × 10^8^ CFU mL^−1^ and LOD of 3 CFU mL^−1^	[85]
	Cu_3_(BTC)_2_-PANI	High sensitivity, LOD of 2 CFU mL^−1,^ and short answer time of 2 min	[86]
Silica NPs	SNP-RB	Wide detection range of 10–10^5^ CFU mL^−1^ and LOD of 8 CFU mL^−1^	[87]
	DNA-HSMs	Wide detection range of 1 × 10^−10–^1 × 10^−5^ µM with R^2^ of 0.982 and LOD of 1.95 × 10^−15^ µM	[88]
Magnetic NPs	Antibody-MNBs	No need for pre-enrichment, LOD of 10^4.45^ CFU mL^−1^ that equal to 1400 bacterial 25 μL and response time less than 60 min	[89]
	Antibody-Fe_3_O_4_	An LOD of 7.4 × 10^4^ and 8.0 × 10^5^ CFU ml^−1^ in pure culture and ground beef samples	[90]
	Fe_3_O_4_@ gold	No need for pre-enrichment and LOD of <1 log CFU mL^−1^	[91]
ZnO NPs	Antibody-piezoelectric ZnO	Wide linear detection of 10^3^–10^7^ CFU mL^−1^ and LOD of 10^3^ CFU mL^−1^	[92]
	DNA-ZnO Nanorod	An LOD of 1.0 × 10^2^ CFU mL^−1^ for target ssDNA of *E. coli*	[93]

LOD: Limit of Detection, LOQ: Limit of Quantitation, CFU: Colony-Forming Unit, NPs: Nanoparticles, MoS_2_: Molybdenum disulphide, Zn-Te: Zinc-Tellurium, CdS: Cadmium Sulphide, BC-Ni: Boron-Carbon-Nickel nanorods, POE: Polyethyleneimine, SWCNTs: Single-Walled Carbon Nanotubes, MOF: Metal–Organic Frameworks, Tb: Terbium, BTC: 1,3,5-benzenetricarboxylic acid, Cu: Copper, PANI: Polyaniline, SNP-RB: Fluorescent Silica NPs, HSMs: Hollow Silica Microspheres, MNBs: Magnetic Nanobeads.

**Table 2:** Different types of nanocarriers for mediating anti-bacterial action against *E. coli.*

No.	Nanocarriers	Drug	Size (nm)	Action	Ref.
A.	Polymeric nanocarriers				
	1. Chitosan nanocarrier	Nettle oil	208.3–369.4	Inhibit *E. coli* (zone of inhibition: 4.11–3.95 cm)	[103]
	2. Chitosan nanocomposites	Cranberry proanthocyanin	122.8 to 618.7	Agglutination and inhibition of *E. coli* to invade epithelial cells	[104]
	3. Mannosylated chitosan	------	180 ± 5	MIC 17.91 μg/mL against *E. coli*, treatment of acute cystitis in mice	[105]
	4. Surface charge conversion nanocarrier	Chlorin e6 (Ce6)	80.9 to 181.8	Inhibit resistant *E. coli* strain and other bacteria, antibiofilm	[106]
	5. Multifunctional PLGA NPs	Caffeic acid phenethyl and juglone	151 to 196	Synergistic effect in eliminating *E. coli*, *S. aureus*, and *leishmania*	[107]
	6. Cationic acrylate copolyvidone NPs	Iodine	~200	Inhibit *E. coli* (99% efficiency) and *S. aureus*	[108]
	7. PCL NPs	Chlorhexidine	152 ± 37	Prevent *E. coli* growth and proliferation	[109]
B.	Lipidic nanocarriers				
	1. NLCs	Ceftriaxone	86	Inhibit *E. coli*	[110]
	2. SLNs	Carvacrol	14.9–25.3	Inhibit *E. coli* and *S. aureus*	[111]
	3. DPPC liposomes	BSP16Lys endolysin	303	Reduced *E. coli* CFU/mL	[112]
	4. DODAB lipidic vesicle/disk	Gramicidin	61–247	Kill *E. coli*	[113]
	5. Rhamnosomes nanovesicles	Nisin	209 ± 4	Activity against *E. coli*, *Listeria monocytogenes*, *S. aureus*, and *P. aeruginosa* biofilms	[124]
C.	Metallic NPs				
	1. Iron oxide NPs	------	10.64 ± 4.73	Retard growth of *E. coli* antibiotic-resistant strains	[116]
	2. Magnetite NPs	------	19	MIC 2.8 µg/mL against *E. coli*, biocompatible to organs	[56]
	3. ZnO NPs	Plant extracts	20–47	Activity against *E. coli* and *S. aureus*	[51]
	4. Ag NPs	------	30	*E. coli* inhibition and sterilization	[117]
	5. Ag NPs	------	8 ± 4	*E. coli* inhibition	[118]
D.	Magnetic nanoparticles				
	BaFe_12_O_19_/xCoFe_2_O_4_	------	71–91	Suitable saturation magnetization and magnetic coercivity. Effective against bacterial (*E. coli*) and fungal strains	[119]
E.	Hybrid nanoparticles				
	1. Polymer-lipid (chitosan-glycerol monostearate) NPs	Ceftriaxone	188–720	Higher mortality rate of *E. coli*	[114]
	2. Chitosan coated nanostructured lipidic carriers (NLCs)	------	292.9 ± 2.5	Inhibit *E. coli* biofilm formation on catheter	[115]
F.	Niosomal gel	Simvastatin	168	Inhibit *E. coli* and *S. aureus*	[120]
G.	Nanoemulsion				
	1. ε-polylysine nanoemulsion	D-limonene	12.21–15.65	Strong synergistic action against *E. coli*, *Bacillus subtilis*, *S. aureus* etc.	[121]
	2. Sodium caseinate based self-assembled nanoemulsion	Thyme oil	90–200	Antibacterial activity against *E. coli* and *S. aureus*	[122]
H.	Bioinspired peptide-based dendrimers	------	------	Activity against clinical isolates of antibiotic-resistant ESBL-producing and MDR isolates of *E. coli*	[123]

## Data Availability

Not Applicable.
