# Correction: Barani et al. Nanomaterials in the Management of Gram-Negative Bacterial Infections. *Nanomaterials* 2021, *11*, 2535

**DOI:** 10.3390/nano16110641

**Published:** 2026-05-22

**Authors:** Mahmood Barani, Mahira Zeeshan, Davood Kalantar-Neyestanaki, Muhammad Asim Farooq, Abbas Rahdar, Niraj Kumar Jha, Saman Sargazi, Piyush Kumar Gupta, Vijay Kumar Thakur

**Affiliations:** 1 Medical Mycology and Bacteriology Research Center, Kerman University of Medical Sciences, Kerman 7616913555, Iran; mahmoodbarani7@gmail.com (M.B.); d.kalantar@kmu.ac.ir (D.K.-N.); 2 Department of Pharmacy, Faculty of Biological Sciences, Quaid-i-Azam University, Islamabad 45320, Pakistan; mz1712@yahoo.com; 3 Department of Medical Microbiology (Bacteriology and Virology), Afzalipour Faculty of Medicine, Kerman University of Medical Sciences, Kerman 7616913555, Iran; 4 Faculty of Pharmacy, Department of Pharmaceutics, The University of Lahore, Lahore 54000, Pakistan; pharma1154@yahoo.com; 5 Department of Physics, University of Zabol, Zabol 9861335856, Iran; 6 Department of Biotechnology, School of Engineering and Technology, Sharda University, Greater Noida 201310, India; nirajkumarjha2011@gmail.com; 7 Cellular and Molecular Research Center, Research Institute of Cellular and Molecular Sciences in Infectious Diseases, Zahedan University of Medical Sciences, Zahedan 9816743463, Iran; sgz.biomed@gmail.com; 8 Department of Life Sciences, School of Basic Sciences and Research, Sharda University, Greater Noida 201310, India; 9 Biorefining and Advanced Materials Research Center, SRUC, Edinburgh EH9 3JG, UK; 10 Department of Mechanical Engineering, School of Engineering, Shiv Nadar University, Greater Noida 201314, India; 11 School of Engineering, University of Petroleum & Energy Studies (UPES), Dehradun 248007, India

In the original publication [1], the citation referring to References 10, 41, 61, 62, 63, 133, 134, and 135 included errors. References 41, 61, 62, and 63 were removed, and references 10, 133, 134, and 135 were replaced with the following references:

10. Breijyeh, Z.; Jubeh, B.; Karaman, R. Resistance of Gram-Negative Bacteria to Current Antibacterial Agents and Approaches to Resolve It. *Molecules* **2020**, *6*, 1340.
133. O’Sullivan, S.; Ali, Z.; Jiang, X.; Abdolvand, R.; Ünlü, M.S.; Plácido da Silva, H.; Baca, J.T.; Kim, B.; Scott, S.; Sajid, M.I.; et al. Developments in Transduction, Connectivity and AI/Machine Learning for Point-of-Care Testing. *Sensors* **2019**, *19*, 1917.
134. Angehrn, Z.; Haldna, L.; Zandvliet, A.S.; Gil Berglund, E.; Zeeuw, J.; Amzal, B.; Cheung, S.Y.A.; Polasek, T.M.; Pfister, M.; Kerbusch, T.; et al. Artificial Intelligence and Machine Learning Applied at the Point of Care. *Front. Pharmacol.* **2020**, *11*, 759.
135. Berg, B.; Cortazar, B.; Tseng, D.; Ozkan, H.; Feng, S.; Wei, Q.; Chan, R.Y.L.; Burbano, J.; Farooqui, Q.; Lewinski, M.; et al. Cellphone-Based Hand-Held Microplate Reader for Point-of-Care Testing of Enzyme-Linked Immunosorbent Assays. *ACS Publ.* **2015**, *9*, 7857–7866.

With this correction, the order of some references has been adjusted accordingly.

The authors state that the scientific conclusions are unaffected. This correction was approved by the Academic Editor. The original publication has also been updated.
